# A Leptospirosis Outbreak in a Riding Stable in Central Italy

**DOI:** 10.3390/vetsci13080750

**Published:** 2026-07-28

**Authors:** Silva Costarelli, Mario D’Incau, Deborah Cruciani, Carmen Panzieri, Alessandro Fiorucci, Sergio Scorcelletti, Alessandro Nunzi, Maria Grazia Brancaleoni, Luca Latini, Antonella Catalano

**Affiliations:** 1 Istituto Zooprofilattico Sperimentale dell’Umbria e delle Marche “Togo Rosati”, 06126 Perugia, Italy; s.costarelli@izsum.it (S.C.);; 2 Centro di Referenza Nazionale per la Leptospirosi, Istituto Zooprofilattico Sperimentale della Lombardia e dell’Emilia Romagna “Bruno Ubertini”, 25124 Brescia, Italy; 3 Servizio di Sanità Animale, USL Umbria 1, 06127 Perugia, Italy; 4 Servizio di Prevenzione, Igiene e Sanità Pubblica, USL Umbria 1, 06127 Perugia, Italy

**Keywords:** equine uveitis, horse, Italy, *Leptospira* spp., microscopic agglutination test, One Health, outbreak investigation, zoonosis

## Abstract

Leptospirosis is a widespread zoonotic disease of major concern for both veterinary and public health. In horses, it is associated with equine recurrent uveitis, a painful condition that may lead to blindness and affects approximately 10% of the global horse population. In May 2025, following the occurrence of three uveitis cases in a riding stable in Central Italy, 94 equine blood samples were collected and tested using the microscopic agglutination test (MAT) for *Leptospira* spp. Antibody titres against serogroup Icterohaemorrhagiae were detected in 68 horses (72.4%). An on-site investigation was conducted, and control measures were implemented, including movement restrictions, clinical monitoring of horses, and antibiotic treatment. Follow-up serological testing showed no significant variation in antibody titres, no additional clinical cases were observed in animals, and no symptoms were reported among individuals exposed to the facility. These findings suggest widespread exposure to *Leptospira* spp. without evidence of ongoing transmission or public health impact. This study highlights the diagnostic and management challenges of equine leptospirosis, together with the importance of surveillance within a One Health framework.

## Simple Summary

Leptospirosis is an infectious disease of domestic and wild animal species. It could be transmitted to humans through direct or indirect contact with infected animals, representing a widespread zoonotic disease. Infected horses may exhibit significant reproductive disorders and a painful condition commonly called “moon blindness”, that may lead to blindness. This study describes an equine leptospirosis outbreak in a riding stable in Central Italy. The diagnosis was based on clinical signs and confirmed by laboratory evidence showing 72.4% of seropositivity among the 94 housed horses. A microscopic agglutination test (MAT) identified antibodies against serogroup Icterohaemorrhagiae of *Leptospira* spp. Consequently, movement restrictions and other control measures were adopted to avoid infection spreading. The outbreak was managed through a One Health approach: collaboration between veterinary and human public health authorities was successful considering that no additional clinical cases were observed in animals and no symptoms were reported among individuals exposed to the facility. Public health was restored in an acceptable timeframe, also favouring the facility owner in terms of safe reopening.

## 1. Introduction

Leptospirosis is recognized as a widespread zoonotic disease, caused by spirochetes according to the List of Prokaryotic names with Standing in Nomenclature [1], belonging to the genus *Leptospira* (family *Leptospiraceae*, order *Leptospirales*) [2]. Given that transmission to humans may occur through direct or indirect contact with infected animals or contaminated environments, leptospirosis represents a significant concern for both veterinary and public health. In humans, it may result in considerable morbidity among susceptible populations [3]. The early symptoms of leptospirosis such as fever, muscle pain, and headache mimic other febrile illness, but could evolve in multiorgan dysfunction involving the liver, kidney, lungs, and/or central nervous system [4,5]. In severe cases, fatality rate is about 5–15% [6]; surviving patients could exhibit chronic postleptospirosis issues, including fatigue, myalgia, malaise, persistent headaches, and visual disturbances [7] with the greatest burden in marginalized communities. This particularly occurs in disadvantaged rural and urban areas of tropical regions, and for this reason the U.S. Centers for Disease Control (CDC) has designated leptospirosis as a neglected tropical disease (NTD) [8].

Horses play a relatively minor role in the spread of the disease compared with rats, other synanthropic rodents, livestock, and wild animals [4,9]. However, leptospirosis in horses may also cause significant reproductive disorders, including placentitis, abortion, and stillbirth, resulting in considerable economic losses [10]. Moreover, *Leptospira* spp. in horses may also cause equine uveitis, a highly painful condition characterized by lacrimation, photophobia, blepharospasm, eyelid and corneal edema, and conjunctival swelling. It is also known as “moon blindness” or “periodic ophthalmia”, has an estimated worldwide prevalence of approximately 10% in the general equine population [11], and represents one of the leading causes of blindness in equids.

This investigation was performed following the occurrence of three cases of uveitis in a riding stable, which prompted the attending veterinarian to implement a sanitary protocol as suggested by the Italian Republic Legislative Decree No. 191 of 4 April 2006—which is the implementation of Directive 2003/99/EC on the Monitoring of Zoonoses and Zoonotic Agents [12]—and according to the Commission Delegated Regulations (EU) 2020/687 [13] and 2020/689 [14].

## 2. Materials and Methods

In May 2025, three cases of equine uveitis were reported at a riding stable in Central Italy. The facility housed 94 horses, comprising 50 mares and 44 geldings, actively engaged in show jumping or recreational activities. The age of the horses ranged from 1 to 25 years, and the majority of them were of mixed breed. Additional demographic information of the study population is presented in Table 1.

Blood samples were collected from all horses by the attending veterinarian and conferred to the Istituto Zooprofilattico Sperimentale dell’Umbria e delle Marche “Togo Rosati” (IZSUM) for serological testing for *Leptospira* spp. The decision to investigate leptospirosis was prompted not only by the presence of uveitis but also by the preliminary information indicating that several abortions had previously occurred on the farm.

After serum separation, the samples were immediately analyzed by the microscopic agglutination test (MAT) using a panel of the eight *Leptospira* serogroups considered epidemiologically relevant to the study area based on previous literature reports [15,16,17]: Icterohaemorrhagiae (s.g. Icterohaemorrhagiae), Pomona (s.g. Pomona), Bratislava (s.g. Australis), Ballum (s.g. Ballum), Hardjo (s.g. Sejroe), Tarassovi (s.g. Tarassovi), Canicola (s.g. Canicola), and Grippotyphosa (s.g. Grippotyphosa). The test was performed starting from an initial serum dilution of 1:100 up to 1:1600, in accordance with the WOAH protocol [18]. Sera with antibody titres equal to or greater than 1:100 were considered positive.

Given that none of the horses had been vaccinated against *Leptospira* spp., the diagnostic findings prompted immediate notification to the local competent veterinary authority (LCVA), which immediately carried out an inspection of the riding stable involved and established that the 94 horses belonged to 42 different owners. All animals were clinically examined and evidence of clinical signs of unilateral and bilateral uveitis, compatible with Leptospirosis, were observed in two horses and one horse, respectively. Furthermore, the clinical history confirmed that some mares had experienced abortions during the previous year, although no additional relevant information could be obtained. The LCVA immediately placed the farm under seizure and imposed movement restrictions on both animals and people: an official ban on the introduction and removal of animals from the facility was imposed together with control measures applied to riding stable visitors. Therefore, visitors were advised to be alert for the onset of clinical signs consistent with leptospirosis, including fever, myalgia, arthralgia, and fatigue. The only worker responsible for animal care at the riding stable underwent both clinical monitoring and serological testing.

In addition, antibiotic therapy consisting of intramuscular procaine benzylpenicillin (9.000 IU/kg) and dihydrostreptomycin (11.25 mg/kg) was administered to all animals for seven consecutive days.

**Figure 1:**
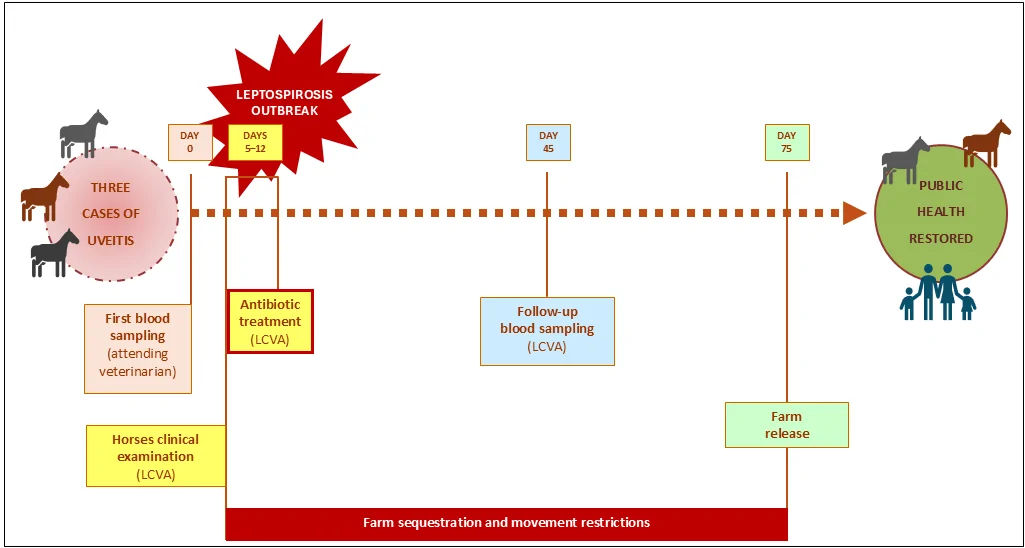
Timeline of events leading to the restoration of public health in the riding stable. LCVA: local competent veterinary authority.

The LCVA finally scheduled follow-up serological testing 30 days after antibiotic therapy treatment (Figure 1).

## 3. Results

The MAT results of the first sampling on the 94 animals are reported in Table 2.

A total of 68 horses (72.4%) were seropositive for *Leptospira* serogroup Icterohaemorrhagiae, with MAT titres of 1:100 (*n* = 20, 21.3%), 1:200 (*n* = 42, 44.7%), and 1:800 (*n* = 6, 6.4%). Among the six horses with the highest antibody titres, three were those affected by uveitis, two animals also tested positive for *L. interrogans* serovar Pomona (1:400), and two for *L. interrogans* serovar Bratislava (1:400). No seropositivity was detected against any of the other serogroups tested. The remaining 26 horses (27.6%) tested seronegative.

The comparison results between the first and the second MAT testing are presented in Table 3.

Serological results from the second sampling confirmed the 26 seronegative animals, showed stable antibody titres in 37 horses (*n* = 20 + 15 + 2), showed a decrease in antibody titres in 26 subjects (*n* = 23 + 3), and showed a one-dilution increase in antibody titre in one horse. For technical reasons, four animals were not subjected to the second sampling (Table 3).

During the investigation period, cases of uveitis remained limited to the initial three animals, with no additional subjects affected. No clinical signs were observed among the staff or visitors of the riding facility.

Two and a half months after the first MAT assessment, and based on the recorded findings, the LCVA removed the movement restrictions.

Information provided by the attending veterinarian indicated an initial clinical improvement in the uveitis. To date, no further cases of uveitis have been reported at the riding stable.

## 4. Discussion

This case provided an opportunity to further investigate certain aspects of the course of *Leptospira* infection in the equine species.

Although horses play a relatively minor role in the disease transmission compared with other domestic and wild animals, epidemiological data remain limited and heterogeneous particularly across Italian regions. In Italy, positivities to serogroup Icterohaemorrhagiae were previously described by Tagliabue et al. 2016 [15], Ebani et al. 2012 [16], and by Bertelloni et al. 2019 [17].

According to Article 9 of the Commission Delegated Regulations 2020/689, the attending veterinarian considered in this case the involvement of *Leptospira* because a group of animals showed clinical uveitis, and this hypothesis was confirmed by positive results from MAT in non-vaccinated animals. Consequently, the event was defined as a leptospirosis outbreak.

The attending veterinarian opted for an indirect diagnostic approach because alternative sampling strategies (e.g., PCR from ocular fluids or urine) would have required more time, been more invasive, and incurred higher costs. Furthermore, placental tissues and aborted fetuses were unavailable. The veterinarian was also aware that the PCR assay routinely used at the IZSUM laboratories detects pathogenic *Leptospira* spp. only at the genus level [19] and does not allow discrimination among species, serogroups, or serovars. In contrast, the microscopic agglutination test (MAT) provides valuable epidemiological information by identifying the presumptive infecting serogroup and was therefore considered the most appropriate diagnostic tool for the purposes of this investigation. Among the 94 horses samples, the highest serological titres (1:800) were observed for serogroup Icterohaemorrhagiae in six subjects, three of whom had developed uveitis. Most of the remaining animals tested negative or showed low antibody titres, ranging from 1:100 to 1:200. Additional seropositivities were observed in some subjects for serogroups Australis and Pomona in horses with the highest antibody titres, attributable to cross-reactivity. The possibility that these serogroups contributed to the infection cannot be completely excluded, but the Icterohaemorrhagiae serogroup was considered the most likely infecting serogroup because it consistently elicited the highest antibody titres.

Considering that leptospirosis is a zoonotic disease, and that the riding facility was frequented by several people (e.g., adolescents, children, and individuals with disabilities), the LCVA immediately involved the Human Prevention, Hygiene and Public Health service and the Occupational Safety and Health Service to assess conditions necessary to ensure public health protection. The area where the riding facility was located was characterized by a rural environment. It was noted that the perimeter of the facility contained various materials unrelated to its activity (e.g., tires, cardboard, stacked firewood, and disused agricultural equipment). These materials provided potential access points and suitable habitats for rodents or other wild species. In addition, the on-site inspection by the LCVA revealed the presence of multiple accumulations of rainwater near the drinking trough, as well as in surrounding areas. Furthermore, the facility was located near a wetland environment (drainage canals and ditches) where the presence of coypus (*Myocastor coypus*) was frequently observed. Wild animals, particularly rodents, are recognized as important maintenance hosts of *Leptospira* spp., capable of chronic urinary shedding and environmental contamination, and thus may serve as natural reservoirs for the pathogen. The area was also frequented by wild boar (*Sus scrofa*), a widespread species in the region that increasingly lives in peri-urban and rural landscapes near human activities and infrastructure. Therefore, wild boars have also been hypothesized as potential carriers of *Leptospira* in various environments, contributing to environmental contamination via urine [20]. The co-occurrence of wildlife reservoirs, the presence of natural water accumulations (e.g., standing water), and the environmental temperatures typical of the spring season likely facilitated the persistence of the organism in the environment and may have contributed to the maintenance and transmission of infection within the facility.

After the on-site inspection, the prevention service implemented the following measures: (i) application of a pest control protocol across the entire riding stable area; (ii) removal of unused materials and clutter that could provide shelter and nesting sites for rodents; (iii) secure storage of feed and hay bales in enclosed facilities; (iv) tracing of owners and individuals who had attended the riding stable in the weeks preceding the movement restrictions measures, to verify the absence or presence of clinical signs, including non-specific symptoms (e.g., fever, myalgia, fatigue).

It is widely recognized that treating all animals with antimicrobial therapy is not fully consistent with the principles of antimicrobial stewardship. However, when the initial MAT results became available, the LCVA was unable to reliably interpret the significance of the lower antibody titres or the negative serological results. At that stage, animals with low or negative MAT titres could not be excluded as being infected, as they might have been sampled before seroconversion or during the early phase of antibody development. Therefore, from a public health perspective, the LCVA decided to administer antimicrobial treatment to all animals in the facility while individuals attending the riding center were only monitored for the possible development of clinical signs compatible with leptospirosis. One month after antibiotic treatment, the second MAT assessment revealed no substantial changes: antibody titres remained stable or showed only a slight decrease, except in one case where the titre increased. Based on the authors’ experience, a similar trend has also been observed in cattle, in which antibiotic treatment appears to limit the progression of infection, by reducing *Leptospira* environmental circulation while serological titres tend to decline slowly and may occasionally increase, reflecting the persistence and activity of the immune response in affected animals. Moreover, a potential variability associated with the microscopic visual interpretation of the MAT titres should be considered.

Given that the incubation period of human leptospirosis rarely exceeds 30 days and is typically 5–15 days, the absence of clinical cases among individuals attending the riding facility throughout the two-month closure was considered by the LCVA to provide indirect evidence that the horses were no longer shedding *Leptospira* at the time of the initial investigation. This conclusion was further supported by the serological follow-up, the absence of additional clinical cases in horses (including no new cases of uveitis), and the stability or decline in antibody titres observed in most animals. Collectively, these findings were consistent with the occurrence of a past outbreak rather than ongoing transmission and indicated that the public health risk had been effectively mitigated. Accordingly, two months after the implementation of official movement restrictions, the LCVA lifted all control measures.

**Limitations paragraph.** The main limitation of this study is the absence of direct diagnostic methods to confirm *Leptospira* infection. The exclusive use of the microscopic agglutination test (MAT) was justified by the feasibility of blood sampling in all horses, the unavailability of placental tissues and aborted fetuses, and the presumed timing of infection, which likely occurred several weeks before clinical detection, making MAT the most appropriate diagnostic tool [21]. In addition, the lack of a validated PCR protocol prevented the investigation of environmental water samples during the emergency response. Other limitations include incomplete clinical information, limited follow-up for four affected horses, and the absence of systematic testing of exposed humans. These constraints should be interpreted considering the nature of the study, which was conceived as a case report describing the multidisciplinary veterinary and public health response to a suspected outbreak rather than as a prospectively designed research investigation.

## 5. Conclusions

The described case provided a valuable opportunity for collaboration between veterinary and human public health authorities. Together, these services conducted the on-site investigation and coordinated the response plan. In fact, based on the EU Regulation 2016/429 on transmissible animal health legislation, the management of infectious and diffusive diseases requires an approach based on laboratory outcomes, knowledge of the specific herd, territorial epidemiology, and risk analysis.

Integrating clinical experience with the knowledge of human and veterinary public services, an equine leptospirosis outbreak was managed, ensuring human and animal health and animal welfare and also favoring the facility owner in terms of safe reopening in an acceptable timeframe.

Therefore, this case exemplifies the application of the One Health approach.

## Figures and Tables

**Table 1:** Demographic information of the 94 horses housed at the riding stable.

Horses (*n*)	Sex	Age Range	Breed
50	Mares	1–19 years old	32 Mixed breeds 15 Italian Saddle Horses 2 Belgian Sport Horses 1 Friesian
44	Geldings	1–25 years old	31 Mixed breeds 11 Italian Saddle Horses 2 Belgian Sport Horses

**Table 2:** MAT results of the first blood sampling.

Horses (*n*)	Clinical Signs	MAT	Serogroup
26	None	Negative	Not applicable
20	None	1:100	Icterohaemorrhagiae
42	None	1:200	Icterohaemorrhagiae
6	Uveitis (*n* = 3)	1:800	Icterohaemorrhagiae
**94**			

**Table 3:** Comparison of MAT results between the first and second blood sampling.

Horses (*n*)	Clinical Signs	MAT 1st Sampling	Serogroup	MAT 2nd Sampling	Trend
26	None	Negative	Not applicable	Negative (*n* = 26)	Stable
20	None	1:100	Icterohaemorrhagiae	1:100 (*n* = 20)	Stable
42	None	1:200	Icterohaemorrhagiae	1:200 (*n* = 15) 1:100 (*n* = 23) 1:400 (*n* = 1) N/A * (*n* = 3)	Stable Decreased Increased N/A *
6	Uveitis (*n* = 3)	1:800	Icterohaemorrhagiae	1:800 (*n* = 2) 1:400 (*n* = 3) N/A * (*n* = 1)	Stable Decreased N/A *
**94**					

* N/A: no follow-up available.

## Data Availability

The original contributions presented in this study are included in the article. Further inquiries can be directed to the corresponding author.

## Abbreviations

The following abbreviations are used in this manuscript:

IZSUM	Istituto Zooprofilattico Sperimentale dell’Umbria e delle Marche “Togo Rosati”
LCVA	Local Competent Veterinary Authority
MAT	Microscopic Agglutination Test
NRCL	National Reference Centre for Leptospirosis
PCR	Polymerase Chain Reaction
